# Use and Propensity to Use Substances as Cognitive Enhancers in Italian Medical Students

**DOI:** 10.3390/brainsci8110197

**Published:** 2018-11-09

**Authors:** Marcella Pighi, Giancarlo Pontoni, Arianna Sinisi, Silvia Ferrari, Giorgio Mattei, Luca Pingani, Elena Simoni, Gian Maria Galeazzi

**Affiliations:** 1 Villa Rosa, Private Accreditated Hospital, Modena 41125, Italy; lamarzela@gmail.com (M.P.); elenasimoni83@yahoo.it (E.S.); 2 Psychiatry Section, Psychophysiological Selection Office, Italian Army National Recruitment and Selection Center, Foligno 06034 (PG), Italy; chucky214@hotmail.it; 3 Department of Biomedical, Metabolic and Neural Sciences, University of Modena and Reggio Emilia, Modena 41124, Italy; arianna.sinisi@gmail.com (A.S.); silvia.ferrari@unimore.it (S.F.); giorgiomattei@alice.it (G.M.); 4 Marco Biagi Department of Economics & Marco Biagi Foundation, University of Modena and Reggio Emilia, Modena, 41121 Modena, Italy; 5 Human Resources, Azienda Unità Sanitaria Locale—IRCCS di Reggio Emilia, Reggio Emilia 42122, Italy; luca.pingani@unimore.it

**Keywords:** neuroenhancement, psychostimulants, medical students, college students, cognitive enhancers

## Abstract

International media has paid attention to the use of substances by healthy subjects to enhance cognitive performance. Medical students are liable to use cognitive enhancers (CE) with the aim of improving academic performance. The study explored use and attitudes toward the use of CE in Italian medical students. The authors anonymously surveyed 433 medical students of the University of Modena and Reggio Emilia with an ad hoc 36-items questionnaire. CE were broadly defined as any substance taken with the purpose of improving cognitive functions, from readily available beverages and substances, such as coffee, tea, energy drinks, and supplements to prescription only medication, such as psychostimulants and modafinil. Response rate was 83.8% (*n* = 363). While the majority of the students (74.7%; *n* = 271) said that they had used substances to improve cognitive functions, only 2 students (0.6%) reported the use of prescription-only medications in the last 30 days. Main reasons for not taking prescription-only drugs were concerns about safety and side effects, reported by 83.3% of students (*n* = 295). A positive attitude toward use was held by 60.3% (*n* = 219) subjects. The surveyed Italian medical students used many substances as CE, but this did not seem to apply significantly to psychostimulants. A multivariable analysis showed that the following variables were related to the propensity to use substances as CE: male gender, self-reported memory impairment, concerns about worsening of cognitive performance, lifetime use of at least one illegal substance, use of any substance (both legal or illegal) in the last 30 days.

## 1. Introduction

The term cognitive enhancement (CE) refers to strategies that are thought to improve cognitive performance and thereby to confer an advantage in certain situations [1]. Cognitive enhancers may be readily available substances, such as caffeine and energy drinks, as well as psychostimulants, when used with the aim at improving cognitive functions. It is reported that healthy subjects increasingly resort to use prescription-only medication—especially psychostimulants used to treat attention deficit hyperactivity disorder (e.g., methylphenidate, amphetamine, atomoxetine) and modafinil—with the purpose of enhancing their cognitive performance [2,3].

Recent studies have linked the use of cognitive enhancers to high levels of stress related to performance in populations of college students, including medical students [4,5]. The latter seems a population liable to use cognitive enhancers. In fact, the prevalence of use reported in this population is around 15% in recent studies [4,6].

Moreover, the attention paid by international mass media to this phenomenon is steadily increasing [7]. The attitude toward the use of cognitive enhancers also varies widely in published studies. For example, in one study, 2 out of 3 participants stated that under no circumstances would they use a cognitive enhancer [8]. Alternatively, another study reported a positive attitude toward its use in 90.6% of the subjects enrolled [9]. Little is known about the use and attitudes to use of cognitive enhancing substances among Italian students. In 2012, 34 medical and nursing students and 44 students of other courses attending a Public Health University Degree in Milan, Italy were surveyed [10]. Subjects were not requested to specify which drug they had used. Researchers found that 12% of the medical sample and 16% of the whole sample had taken drugs to enhance their cognitive functions. This rate is not confirmed by a recent cross-sectional study conducted at the University of Verona, Italy [11], showing that among medical students, stimulants use increased from the first academic year (2.9%) to the third (6.5%), and then decreased during the fifth year (1.7%). Such variations may be partly due to the different definitions adopted by each study to define the non-medical use of prescription-only medication. Moreover, such contrasting findings may be a consequence of not specifying the exact substances considered. Therefore, given the methodological diversity, as well as the different purposes for these surveys, results are difficult to compare. For the sake of comparability, it was suggested that future surveys exploring the use of cognitive enhancers should contain a clear definition of which substances are considered cognitive enhancers [3].

The primary aim of this research was to obtain a better estimate of the prevalence of use and attitudes toward the use of psychostimulants and other substances and nutrients with the purpose of CE among a sample of Italian medical students. The secondary aim was to assess factors associated with the use of CE in this population.

## 2. Materials and Methods

After reviewing the literature on CE and contacting researchers in the field [10,12,13], and considering recommendations made by Ragan et al. for research in this field [3], we developed an ad hoc 36 items survey. The choice to develop an ad hoc questionnaire was due to the fact that a standard definition of CE and a related surveying tool are not available [3]. A pilot survey was previously tested on a group of university students not involved in this research, to check for user-friendliness and understandability. For each academic year involved, questionnaires were delivered to all students in the classroom at the beginning of lessons (11:00 a.m.), and then collected at the end (16:00 p.m.). No incentives of any kind were provided to participants. There were no mandatory questions in the questionnaire, therefore students could skip questions. The survey included yes/no, multiple choice, and 7-points Likert-type questions, which were dichotomized before the analysis. We provided subjects with an operational definition of cognitive enhancers at the beginning of the questionnaire (“substances taken to improve one’s cognitive performance—e.g., attention, concentration, memory, intelligence—may be cognitive enhancers”). Cognitive enhancers were broadly defined as any substance taken with the purpose of improving cognitive functions, from readily available beverages and substances, such as coffee, tea, energy drinks, and supplements to prescription-only medication, such as psychostimulants and modafinil. We used a broad definition (which included the use of tea and coffee) because it considers the specific goal of the person in drinking coffee or tea, that is improving cognitive functions, as per instructions. We explicitly asked students about their current and past use of methylphenidate, amphetamine, atomoxetine, and modafinil, but also of common nutrients and dietary supplements taken with the specific goal of improving their cognitive performance. We recorded the timing of use (more than 12 months ago, between one month, and 12 months ago, in the last 30 days), and collected socio-demographic data. We recorded a subjective self-evaluation of respondents’ cognitive function by means of the following question: “How do you rate your performance with respect to: 1. Memory, 2. Attention, 3. Concentration, 3. Intelligence?”. Respondents could indicate seven options, from “very good” to “very scarce” in a Likert-type scale. Additionally, the students’ own perception of the degree of pressure on performance they received in the medical school was also investigated using seven points Likert-type questions. The questionnaire also presented a hypothetical scenario asking subjects if they would use a novel chemical cognitive enhancer without known side effects, if its use would be legal. We also explored students’ knowledge and opinion of the phenomenon of cognitive enhancement.

We distributed the survey to 433 undergraduate medical students of the University of Modena and Reggio Emilia during the Spring term. Descriptive statistics were performed by means of means, medians, standard deviations, and ranges. Inferential statistics were made by means of binary logistic regression. First, all covariables (age, sex, year of study, emotional experience related to study, self-perceived performance, self-assessment of attention, concentration, memory and intelligence, concerns about worsening of cognitive performance, perceived pressure to achieve good academic results, and alcohol and illegal substances use) were dichotomized according to their median, and univariate logistic regressions were performed. Response variables were: Question 16a (”Have you ever used one of the following substances to enhance your cognitive performances?”); Question 16b (“Have you used one of the following substances to enhance your cognitive performances in the last 30 days?”); Question 28 (“Would you use a hypothetical novel chemical cognitive enhancer without known side effects if it had been legally available?”). Then, three multivariate logistic models were built, using the same response variables, and including as explanatory variables only covariates that had reached a *p* < 0.25 level of statistical significance at the univariate analysis, to reduce type II error. In the multivariate regression analysis, the usual level of significance (*p* < 0.05) was set to identify association between covariables.

The study was approved by the Review Board of the Medical Degree Course of the University of Modena and Reggio Emilia.

## 3. Results

Of the 433 medical students surveyed, 363 (83.8%) responded. Mean age was 22.4 years (range 18–48 years). The majority of the subjects were women (55.9%, *n* = 203), and 271 students (74.7%) reported use of substances to improve cognition in the last 30 days, whilst lifetime use was reported by 86.8% (*n* = 315) subjects. Table 1 details lifetime, last year, and last 30 days frequency of use.

We asked subjects if someone had ever suggested to them to take a drug to enhance their cognitive abilities and/or to deal with fatigue and sleepiness and who suggested it. In 87.1% of cases (*n* = 316), the answer was negative, while 21 individuals received this advice from friends, acquaintances, or family members, 16 by a doctor, 9 from a colleague, 7 from a pharmacist, and 1 from others. Only 2 students reported the use of amphetamine in the last 30 days (0.6%), while nobody reported the use of modafinil, methylphenidate, or atomoxetine in the last 30 days. One student (0.3%) reported lifetime use of methylphenidate and 3 students (0.8%) reported lifetime use of amphetamine. Further details on use and attitude toward use of cognitive enhancers are detailed in Table 2.

The majority of students (56.2%, *n* = 204) were indeed worried about a possible decline in cognitive abilities. About a third (29.5%; *n* = 107) felt they were in a stressful condition due to study demands. About a fifth judged their attention (18.2%; *n* = 66), concentration (19.6%; *n* = 71), memory (17.4%; *n* = 63), intelligence (22%; *n* = 80) as unsatisfactory. A significant fraction rated their study results as unsatisfactory in relation to the efforts made (17.4%; *n* = 63). The majority of students (69.7%; *n* = 253) perceived excessive pressure to achieve good academic results from the University environment. More than half (54%; *n* = 196) of the students had already heard about the use of prescription drugs (atomoxetine, amphetamine, methylphenidate, and modafinil) as cognitive enhancers. Half of the students (50.4%, *n* = 183) reported that they did not agree with pharmacological CE in general; a slightly bigger fraction (57.6%; *n* = 209) disagreed with CE to improve academic results. This ratio reversed (46.3%; *n* = 168) if cognitive enhancers were to be used by soldiers engaged in military actions.

Table 3 shows the results of the univariate logistic regressions. The lifetime prevalence of use of any cognitive enhancer was individually associated with poor self-assessed attention, memory (the latter, at a 10% significant level), and perceived performance in at least one amongst attention, memory, intelligence, and concentration. Moreover, it was associated with increased pressure to achieve good academic results, and with the following questions concerning substance misuse: lifetime use of at least one illegal substance (Cannabis, Heroin, Cocaine, Amphetamines, Hallucinogenic), lifetime alcohol use, last year use of any substance (legal or illegal).

With respect to the use of cognitive enhancers in the previous month, it was associated with female gender, poor self-assessed attention and perceived performance in at least one among attention, memory, intelligence, and concentration. It was also associated with lifetime use of at least one illegal substance (Cannabis, Heroin, Cocaine, Amphetamines, Hallucinogenic), lifetime alcohol use, lifetime use of any substance (legal or illegal, at a 10% significance level), last year use of any substance (legal or illegal), and last 30 days use of any substance (legal or illegal).

Finally, with respect to the attitude toward the use of cognitive enhancers, it was associated with male gender, emotional experience related to study (namely, increased self-reported distress), poor self-assessed attention, memory and perceived performance, and increased concerns about self-reported worsening of cognitive performance and perceived pressure to achieve good academic results. As far as substance misuse was concerned, the outcome of the investigation suggested that the positive attitude toward CE was associated with the use of any substance (both legal or illegal, recent or remote).

Table 4 shows the results of the runs of three multiple regression models. Lifetime use of any substance to enhance cognitive performance was associated with perceived pressure to achieve good academic results, self-reported impairment concerning attention, and use of any substance (both legal or illegal) in the last year. The use of cognitive enhancers in the last month was associated with self-reported impairment concerning any cognitive function (attention, memory, etc.), and lifetime use of at least one illegal drug.

With respect to the attitude toward using a hypothetical cognitive enhancer, we found the latter to be associated with male gender, self-reported impairment concerning memory, concern about worsening of cognitive performance, lifetime use of at least one illegal drug, and the use of any substance (both legal or illegal) in the last month.

## 4. Discussion

This study investigated the use and attitudes toward use of psychostimulants as CE in a sample of Italian university students. Our findings showed that the Italian medical students surveyed used many readily available drinks and substances (such as coffee and caffeine) as cognitive enhancers, but this only minimally applied to psychostimulants (i.e., amphetamine, methylphenidate, atomoxetine, and modafinil). Notably, the prevalence of use of the latter substances as cognitive enhancers reported in the literature is generally higher (6.2% among Swiss students [9]; 1.29% among German students [14]; 6.2% for modafinil, among British and Irish students [15]; 11.9% among Australian university students [6]; and 18% among American students [16]). Such differences may be partly due to the accessibility of psychostimulants in the different countries.

In fact, lack of availability is one of the most common reasons reported for why students did not try potential cognitive enhancers [15].

More than half of the students were aware of the concept of pharmacological cognitive enhancement, consistent with other European research [4,9,14,17]. In line with this, a high percentage of students (74.7%, *n* = 271) said that they had used some substances as cognitive enhancers in the last 30 days and 86.8% (*n* = 315) in their lifetime. This prevalence was considerably higher than what was reported in the only two other available Italian studies on this topic [10,11], though not specifically limited to cognitive enhancers. In particular, Castaldi et al. [10] reported a lifetime prevalence of 16%, while Majori et al. [11] reported 11.3%. With respect to readily available licit substances, among all students enrolled to our study, prevalence rates for the use of caffeine and tea were the highest, consistent with available literature [17].

In line with the main reported reason for not using psychostimulants as cognitive enhancers (fear of side effects), the majority (60.3%) of the students in our sample had a positive attitude toward the use of a hypothetical “safe” cognitive enhancer [13]. As the multivariable analysis pointed out, a positive attitude toward the use of cognitive enhancers is associated with male gender, consistent with other studies in the literature [9,18]. In addition, the use of licit/illicit substances in the last month or lifetime was associated with a positive attitude toward cognitive enhancers, as pointed out by other studies [4,9,15]. With respect to the association between positive attitudes toward cognitive enhancers and self-reported cognitive impairment (concerning memory or other cognitive functions), it is worth noting that almost a third of the students in our sample found studying stressful. This may at least partly explain our findings, since it was pointed out that high levels of stress (due to long hours of studying and academic pressure) were associated with the use of cognitive enhancers [4,5,8].

In our study, the degree of perceived pressure to achieve good academic results was associated with the lifetime prevalence of the use of licit cognitive enhancers. These findings warrant attention, and calls for the design of further research studies, in light of the possible interconnections between high levels of distress, substance misuse (both as cognitive enhancers or not), burn-out, and stigma among medical students, as reported by other authors [19,20,21,22].

One strength of the present study is the high response rate (83.8%; *n* = 363). Additionally, this study helped to shed light on a topic seldom addressed by Italian research, though with noticeable implications. Despite this, several limitations need to be acknowledged. First, since only one university was involved, the generalizability of our findings to the whole Italian student population is limited. In fact, different places or regions (urban and rural) may play a major role in the knowledge of psychostimulants use and awareness. We also investigated only medical students. This may limit the translation of our findings to other populations (e.g., non-medical students). Despite this, the study design made the study feasible, and provided interesting findings that may prompt other research on this topic to overcome the limitations reported here. Secondly, in our research, we did not use a standardized instrument, but an ad hoc questionnaire. However, this choice was motivated by what was reported in the literature with respect to methodological aspects concerning this research field [3]. Third, only lifetime use and use in the last 30 days were explored. This does not allow a full assessment of the extent and chronicity of use. Fourth and final, we relied on subjective ratings of the respondents’ own cognitive function and performance pressure, which may be inaccurate. Other studies aiming to overcome all the limitations mentioned herein are currently on our research agenda.

## 5. Conclusions

The students enrolled in our sample were well-aware of the concept of cognitive enhancement. Despite this, we found that the prevalence of use of psychostimulants amongst a sample of Italian medical students was low. We found that a significant fraction of the sample was worried about academic performance and felt stressed possibly because of it. Such sub-groups seem more prone to use cognitive enhancers. This is an issue which warrants attention and calls for further research on the topic.

## Figures and Tables

**Table 1:** Lifetime, last year, last 30 days use of substances as cognitive enhancers by Italian medical students at the University of Modena and Reggio Emilia, Italy.

Substance	Last 30 Days Use (%, *n*)	Last Year Use (%, *n*)	Ever Used (%, *n*)
Any (of the below-listed substances)	74.7%, 271	83.2%, 302	86.8%, 315
Coffee	73.0%, 265	81.0%, 294	82.9%, 301
Tea	33.3%, 121	48.2%, 175	50.7%, 184
Coke, Pepsi	15.7%, 57	22.3%, 81	28.4%, 103
Energy drinks	7.4%, 27	16.0%, 58	24.2%, 88
Vitamins	8.8%, 32	15.2%, 55	20.1%, 73
Tobacco	10.2%, 37	14.0%, 51	15.4%, 56
Omega-3 fish oils	5.2%, 19	8.5%, 31	10.7%, 39
Gingko-biloba	1.7%, 6	5.5%, 20	9.1%, 33
Caffeine tablets	1.7%, 6	5.0%, 18	6.6%, 24
Nasal decongestants	0.8%, 3	1.9%, 7	3.0%, 11
Cholinergic drugs	0.0%, 0	0.8%, 3	1.1%, 4
Amphetamine salts	0.6%, 2	0.8%, 3	0.8%, 3
Methylphenidate	0.0%, 0	0.0%, 0	0.3%, 1
Atomoxetine	0.0%, 0	0.0%, 0	0.0%, 0
Modafinil	0.0%, 0	0.0%, 0	0.0%, 0
Others (e.g., chocolate, arginine, honey, acetylcarnitine, ginseng, etc.)	7.7%, 28	11.6%, 42	12.7%, 46

**Table 2:** Use and reasons for (not) using cognitive enhancers in the sample.

**Have You Ever Used Psychostimulants (i.e., Methylphenidate, Atomoxetine, Modafinil, Amphetamines) as Cognitive Enhancers?**	No (97.5%; *n* = 354), Yes (0.8%; *n* = 0.3), Missing (1.7%; *n* = 0.6)
**What are the reasons why you would not use prescription drugs as cognitive enhancers?**	- I am concerned about safety and side effects (83.3%, *n* = 295); - I feel I do not know enough about such drugs (77.1%, *n* = 273); - I think I do not need them for CE (63.6%; *n* = 225); - I am afraid of becoming addicted (60.5%, *n* = 214); - I want to succeed without the help of a substance (57.1%, *n* = 202); - I am not aware of the use of such substances for this purpose (47.1%, *n* = 167); - I prefer “natural” substances (28.2%, *n* = 100); - I think using a cognitive enhancer amounts to “cheating” (32.8%, *n* = 116); - I am afraid that my use would become known to other people (26.3%, *n* = 93); - I do not think that these substances are effective (20.9%, *n* = 74); - I do not use such substances since they are obtainable only by prescription for specific conditions (19.2%, *n* = 68); - I do not use such substances because of practical difficulties in obtaining the substance (16.1%, *n* = 57); - I am concerned about their cost. (15.3%, *n* = 54).
**Would you use a hypothetical novel chemical cognitive enhancer without known side effects if it had been legally available?**	Yes/Favor (60.3%; *n* = 219)
**If you answered no to the previous question, what is the reason?**	- I think I do not need enhancement (86.2%, *n* = 119); - I want to succeed without the help of substances (74.6%, *n* = 103); - I am afraid of unknown side effects (70.3%, *n* = 97); - I believe that using a cognitive enhancer amounts to “cheating” (52.9%, *n* = 73); - I prefer “natural” substances (29.7%, *n* = 41); - I am afraid that my use would become known to other people (18.1%, *n* = 25).

**Table 3:** Results of univariate logistic regression. Bold *p*-values indicate significant covariables (*p* < 0.25) at the univariate analysis and are therefore included in the multiple analysis.

	Question 16a: Have You Ever Used One of the Following Substances to Enhance Your Cognitive Performances? (0 = Sometimes/Any Substance; 1 = Never)			Question 16b: Have You Used One of the Following Substances to Enhance Your Cognitive Performances in the Last 30 Days? (0 = No; 1 = Yes)			Question 28: Would You Use a Hypothetical Novel Chemical Cognitive Enhancer Without Known Side Effects If It Had Been Legally Available? (0 = No; 1 = Yes)		
	OR	*p*-Value	95% CI	Or	*p*-Value	95% CI	OR	*p*-Value	95% CI
Sociodemographic features									
1. Dichotomous age (>22)	0.83	0.56	0.44–1.56	1.31	0.27	0.80–2.16	1.06	0.72	0.70–1.68
2. Sex (female)	0.84	0.57	0.45–1.53	1.27	**0.02**	1.03–1.56	0.64	**0.04**	0.41–0.99
3. Year (I–VI)	1.09	0.48	0.86–1.39	1.07	0.45	0.89–1.30	1.06	0.53	0.89–1.26
Psychological features									
4. Emotional experience related to study	0.87	**0.22**	0.71–1.08	1.06	0.44	0.91–1.25	1.34	**<0.01**	1.15–1.57
5. Self-perceived performance	0.88	**0.24**	0.70–1.09	1.06	0.52	0.89–1.25	1.24	**<0.01**	1.06–1.46
6. Attention self-assessment	0.76	**0.02**	0.60–0.96	1.20	**0.04**	1.01–1.43	1.21	**0.02**	1.04–1.42
7. Concentration self-assessment	0.82	**0.11**	0.65–1.04	1.08	0.38	0.90–1.29	1.01	**0.22**	0.94–1.30
8. Memory self-assessment	0.80	**0.07**	0.64–1.01	1.07	0.44	0.90–1.27	1.50	**<0.01**	1.27–1.79
9. Intelligence self-assessment	0.94	0.65	0.71–1.23	0.96	0.70	0.78–1.18	1.01	0.88	0.84–1.23
10. Low perceived performance in at least one among attention, memory, intelligence, concentration (score of 1–2 in a 7-points Likert scale)	0.29	**0.04**	0.09–0.98	2.55	**0.02**	1.16–5.60	1.2	0.57	0.68–2.15
11. Concern about worsening of cognitive performance	1.06	0.47	0.90–1.26	1.03	0.63	0.91–1.18	1.28	**<0.1**	1.13–1.44
12. Perceived pressure to achieve good academic results	0.84	**0.04**	0.72–0.99	1.08	**0.23**	0.95–1.23	1.14	**0.03**	1.01–1.29
Substances use									
13. Lifetime use of at least one illegal substance (Cannabis, Heroin, Cocaine, Amphetamines, Hallucinogenic)	0.44	**0.03**	0.21–0.92	2.35	**<0.01**	1.35–4.08	3.39	**<0.01**	2.06–5.58
14. Lifetime Alcohol use	0.32	**<0.01**	0.14–0.73	2.29	**0.03**	1.10–4.76	3.01	**<0.01**	1.45–6.26
15. Lifetime use of any substance (legal or illegal)	0.44	**0.10**	1.17–1.18	2.12	**0.08**	0.91–4.91	2.65	**0.02**	1.15–6.09
16. Last year use of any substance (legal or illegal)	0.34	**<0.01**	0.17–0.69	2.32	**<0.01**	1.25–4.32	2.17	**0.03**	1.19–3.94
17. Last 30 days use of any substance (legal or illegal)	0.75	0.33	0.38–1.39	1.94	**0.01**	1.17–3.23	2.52	**<0.01**	1.57–4.04

**Table 4:** Results of the multiple binary logistic regressions.

**Question 16a: Have You Ever Used One of the Following Substances to Enhance Your Cognitive Performances? (0 = Sometimes/Any Substance; 1 = Never)**	**OR**	**Std. Err.**	***p*** **-Value**	**95% CI**
Perceived pressure to achieve good academic results	0.82	0.07	0.03	0.69–0.93
Attention self-assessment (each degree of low perceived performance on a 1–7 points Likert scale)	0.78	0.99	0.05	0.61–1.00
Last year use of any substance (legal or illegal)	0.29	0.11	<0.01	0.14–0.60
**Question 16b: Have you used one of the following substances to enhance your cognitive performances in the last 30 days? (0 = no; 1 = yes)**	**OR**	**Std. Err.**	***p*** **-Value**	**95% CI**
Low perceived performance in at least one among attention, memory, intelligence, concentration (score of 1–2 in a 7-points Likert scale)	2.80	1.19	0.01	1.22–6.45
Lifetime use of at least one illegal substance (Cannabis, Heroin, Cocaine, Amphetamines, Hallucinogenic)	2.23	0.63	<0.01	1.28–3.90
**Question 28: Would you use a hypothetical novel chemical cognitive enhancer without known side effects if it had been legally available? (0 = no; 1 = yes)**	**OR**	**Std. Err.**	***p*-Value**	**95% CI**
Sex (female)	0.59	0.15	0.04	0.36–0.97
Memory self-assessment (each degree of low perceived performance on a 1–7 points Likert scale)	1.46	0.14	<0.01	1.21–1.77
Concern about worsening of cognitive performance (each degree of concern on a 1–7 points Likert scale)	1.19	0.08	0.01	1.04–1.37
Lifetime use of at least one illegal substance (Cannabis, Heroin, Cocaine, Amphetamines, Hallucinogenic)	2.48	0.71	<0.01	1.41–4.35
Last 30 days use of any substance (legal or illegal)	2.15	0.60	<0.01	1.24–3.72
